# Barriers to SGLT2 inhibitor prescribing in primary care: findings from a cross-sectional questionnaire study in England

**DOI:** 10.1093/ckj/sfag144

**Published:** 2026-05-06

**Authors:** Rakesh Dattani, Neville Purssell, Raakhee Desilva, Madhvi Joshi, Andrew H Frankel, Hutan Ashrafian, Ara Darzi, Frederick W K Tam

**Affiliations:** Institute Global Health Innovation, Imperial College London, London, UK; West London Renal and Transplant Centre, Imperial College NHS Trust, London, UK; North West London Integrated Care Board, London, UK; North West London Integrated Care Board, London, UK; Harrow Collaborative PCN, London, UK; West London Renal and Transplant Centre, Imperial College NHS Trust, London, UK; Institute Global Health Innovation, Imperial College London, London, UK; Institute Global Health Innovation, Imperial College London, London, UK; Centre for Inflammatory Disease, Department of Inflammation and Immunology, Imperial College London, London, UK

To the Editor,

Sodium–glucose cotransporter-2 inhibitors (SGLT2i) reduce progression of chronic kidney disease (CKD) and cardiovascular risk across both diabetic and nondiabetic populations [1–3]. They are now recommended within national and international guidelines [4, 5]. Despite this, their uptake in primary care remains variable. The aim of this study was to explore barriers to SGLT2i prescribing in primary care, and to understand how these sit within the wider context of CKD screening, coding, and management.

We conducted a cross-sectional questionnaire study of primary care healthcare professionals in England to explore barriers to SGLT2i prescribing within the context of routine CKD management. A total of 109 complete responses were analysed, predominantly from general practitioners, with representation across multiple Integrated Care Boards.

Most respondents recognized the role of SGLT2i in cardiovascular and renal risk reduction, with 76.1% supporting their use in high-risk patients with type 2 diabetes and 75.2% supporting use in nondiabetic populations where licensed. However, confidence in prescribing was mixed, with only 48.0% reporting feeling somewhat or extremely comfortable initiating SGLT2i, while 32.3% reported feeling uncomfortable. These findings suggest that variation in prescribing is driven less by uncertainty around therapeutic benefit and more by challenges in practical implementation.

The most commonly reported barrier was lack of experience (38.5%), followed by uncertainty regarding indications, contraindications and monitoring requirements (26.6%), and limited time for patient counselling (18.3%). A smaller proportion of respondents felt that SGLT2i should be initiated in secondary care. Taken together, these findings indicate that SGLT2i continue to be perceived by some clinicians as relatively specialist therapies, despite increasing use across multiple long-term conditions (Table 1).

**Table 1:** Knowledge, confidence, barriers, and enablers for SGLT2 inhibitor prescribing. CVD, cardiovascular disease; T2DM, type 2 diabetes mellitus; uACR, urinary albumin-to-creatinine ratio; eGFR, estimated glomerular filtration rate.

Knowledge and attitudes	
	Response (%)
Use for CVD in T2DM	76.1
Use in T2DM (uACR > 25)	58.7
Use in nondiabetic CKD	75.2
Aware glycaemic effect varies with eGFR	47.7
Unsure about glycaemic effect	41.3
**Confidence in prescribing**	
	Response (%)
Comfortable (somewhat + extremely)	48
Uncomfortable (somewhat + extremely)	32.3
Neutral	19.6
**Barriers to prescribing**	
	Response (%)
Lack of experience	38.5
Uncertainty (indications/monitoring)	26.6
Time constraints for counselling	18.3
Prefer initiation in secondary care	9.2
**Enablers to prescribing**	
	Response (%)
Clear guidance (by indication/eGFR)	55.9
Patient education materials	48.6
Greater secondary care support	33.9
Educational sessions	23.9
**System-level support (general CKD care)**	
	Response (%)
CKD dashboard	47.7
Shared care model	41.3
Better integration primary–secondary care	38.5
Financial incentives	37.6

Importantly, these barriers occur alongside wider variation in CKD care. While most respondents reported annual screening of at-risk individuals, approaches to urine albumin-to-creatinine ratio testing were inconsistent, and CKD coding was frequently limited to more advanced stages of disease. This may reduce opportunities to identify patients earlier in the disease trajectory and initiate kidney-protective therapies at a stage where benefit may be greatest.

Encouragingly, respondents identified clear and practical enablers. The most frequently cited were access to concise prescribing guidance based on indication and kidney function, availability of patient facing educational materials, and improved access to secondary care advice. These findings suggest that primary care clinicians are willing to prescribe SGLT2i but require greater support to do so confidently within routine, time-limited consultations.

The widespread adoption of renin–angiotensin–aldosterone system inhibitors in CKD provides a useful precedent. With appropriate guidance, system support, and familiarity, primary care has demonstrated the ability to deliver kidney-protective therapies effectively. In this context, variation in SGLT2i prescribing is likely to reflect their current position along the implementation pathway rather than a limitation in clinician capability.

Interventions to improve uptake should therefore focus on practical implementation. Embedding simple decision support tools within clinical systems, aligning prescribing prompts with CKD coding, and strengthening integration between primary and secondary care may support more consistent use. Earlier and wider adoption of SGLT2i has the potential to reduce progression to advanced CKD, with downstream benefits including reduced need for dialysis and transplantation.

In summary, variation in SGLT2i prescribing in primary care appears to be driven by uncertainty around practical implementation rather than lack of awareness of benefit. Addressing this gap through clear guidance, decision support, and improved integration between primary and secondary care may support earlier and more consistent use of these therapies. This represents an important opportunity to reduce progression of CKD and improve long-term patient outcomes.
